# Calling on the Client’s Perceptions About the Contributions of Physiotherapists Working in Occupational Health Services

**DOI:** 10.1177/2374373519831447

**Published:** 2019-02-22

**Authors:** Laran Chetty

**Affiliations:** 1 Occupational Health and Wellbeing Centre, Royal Free London NHS Foundation Trust, London, United Kingdom

**Keywords:** physiotherapy, occupational health, contribution, clients

## Abstract

**Background:**

Clients are the recipients of occupational health care. To date, little is known about the perceptions of clients about the contribution of physiotherapists working in occupational health services. Gathering this information is imperative to understanding and responding to clients’ needs.

**Methods:**

An interpretative qualitative study was undertaken and face-to-face interviews were conducted with clients from 2 occupational health services situated within the National Health Service in the United Kingdom. Data were tape-recorded and transcribed verbatim in full. Data were analyzed using thematic analysis.

**Results:**

There were 9 clients interviewed, predominantly comprising secretaries, staff nurses, and care assistants. Thematic analysis revealed 2 main themes: vocational rehabilitation and health promotion. Clients revealed distinct subcomponents of the contribution of physiotherapists in occupational health services such as functional capacity evaluations, job demand analysis, and work-specific rehabilitation. Promoting staff health was another pertinent issue reported by clients.

**Conclusions:**

Clients clearly felt that physiotherapists made a contribution to occupational health services. This information lays the groundwork for the development of physiotherapists within occupational health services, which in turn will help clients achieve better care and health outcomes.

## Introduction

Physiotherapists are often referred to as an allied heath member within a cluster of other professions. Traditionally, physiotherapists assess, diagnose, and treat musculoskeletal, respiratory, and neurological disorders with the goal of restoring functional independence and improving quality of life (1,2). However, the role and responsibilities of the physiotherapist is continually expanding to different clinical settings because the skill base of the profession allows for independent clinical reasoning within multiple settings (3).

Within the occupational health setting, physiotherapists are focused on improving the health and well-being of people at work with a commitment of care focused toward the biopsychosocial model (1,2). The paradigm shift in occupational health in the National Health Service in the United Kingdom over the past decade has contributed to physiotherapists gaining recognition within occupational health services (4,5). Since 2010, the occupational health accreditation process specified that occupational health physiotherapists gather the perceptions of clients in order to understand their needs and to determine to what extent it was being met. To date, little is known about the perceptions of clients about the contribution of physiotherapists working in occupational health services. Gathering this information is imperative to understanding and responding to clients’ needs and could be used to inform the development of the role and responsibilities of physiotherapists working in occupational health services.

The aim of the study was therefore to explore the perceptions of clients about the contribution of physiotherapists within occupational health services.

## Methods

An interpretative qualitative study design was undertaken and face-to-face interviews were conducted at 2 occupational health departments situated within the National Health Service, United Kingdom. Two occupational health departments were strategically chosen in order to eliminate the effects of coercion and conflicts of interest. The two occupational health departments chosen comprised multidisciplinary professional groups and included occupational health physicians, nurses, physiotherapists, and psychologists.

Clients were recruited through recruitment pamphlets placed in the reception area of each occupational health department. Clients who contacted the researcher expressing an interest in taking part in the study were sent a participant consent form. Clients were excluded if they were unwilling or unable, for any reason, to give their written consent. Semi-structured interviews were conducted with clients of different characteristics (Table 1). Semi-structured interview is a managed verbal exchange allowing areas of interest to the researcher to be covered, with the flexibility to allow participants to freely expand on areas if they wished to do so.

**Table 1.** Characteristics of Clients.

Site	Occupation	Gender	Appointments	Employment Status	Age Range	Ethnicity	Serious Health Problems/Disability
Hospital 1	Care assistant	Female	6 sessions	Full-time	40-55	BAME^a^	Yes
	Domestic	Female	8 sessions	Full-time	40-55	BAME^a^	Yes
	Coordinator	Male	4 sessions	Full-time	25-40	White	No
	Staff nurse	Female	12 sessions	Full-time	40-55	White	Yes
	Secretary	Female	2 sessions	Full-time	40-55	White	No
Hospital 2	Care assistant	Female	3 sessions	Full-time	25-40	BAME^a^	Yes
	Secretary	Female	4 sessions	Full-time	20-35	White	No
	Secretary	Female	4 sessions	Full-time	40-55	White	No
	Staff nurse	Female	8 sessions	Part-time	40-55	White	Yes

^a^Black, Asian, minority ethnic.

During the interviews, clients were asked about their experiences with the physiotherapist, the kind of services physiotherapists provide, and their views about how physiotherapists contribute to occupational health services. Medical jargon was avoided so that participants felt familiar and comfortable with the language used and to put them at ease. Data collection continued until the researcher felt that data saturation was reached, that is, no new relevant data were found. Interviews were tape-recorded and transcribed verbatim by the researcher in full.

Thematic analysis was used to analyze the data. It is a process of identifying, coding, and categorizing the primary patterns of data. The transcriptions were carefully read repeatedly, with initial ideas being noted. A list of all ideas was made and similar topics were coded and grouped together to form the main themes. Topics not forming part of the main themes were refined into relevant subcategories.

To ensure research trustworthiness, a second reviewer with expertise in qualitative data analysis independently reviewed the data and any discrepancies in interpretation were resolved by discussion. The study was granted ethical approval by Middlesex University London Ethics committee in January 2016. Research governance approval was obtained at each National Health Service site before commencing data collection.

## Results

There were 9 clients who included 3 secretaries, 2 staff nurses, 2 care assistants, 1 coordinator, and 1 domestic (Table 1). Two main themes and 7 subcategories were identified (Table 2). A summary of quotes is provided under each subcategory (Table 3).

**Table 2.** List of Themes and Subcategories.

Theme 1	Vocational rehabilitation
Subcategories	Functional capacity evaluations
	Job demand analysis
	Work-specific rehabilitation
	Support for injuries at work
Theme 2	Health promotion
Subcategories	Improving staff health
	Job coaching
	Development of job descriptions

**Table 3.** Summary of Quotes Under Subcategories.

Functional capacity evaluations
C1: “Sometimes, the employer needs fitness for work assessments even before we start the job. This is where physios play a huge part…they conduct pre-evaluation functional assessments, and give detailed reports about our capabilities for the job.”
C4: “Physios are much better equipped than doctors to compare the physical abilities of clients to functional demands of the job. Unless occupational health doctors have a special interest in functional testing, I don’t think they will go out of their way to evaluate this.”
C1: “Physios have this ability to choose a wide range of functional battery tests based on targeted jobs, and they can also develop tools specific for vocational tasks.”
Job demand analysis
C7: “The physio also evaluates the demands of work and tasks…this is important because overall it helps promote our well-being and fitness.”
C2: “Physios are valuable for identifying and quantifying risk factors associated with a particular job.”
C1: “Following their assessments, they are also able to develop specific job-related adaptation strategies, and this is useful when needing to return to work.”
Work-specific rehabilitation
C5: “Occupational physios focus on developing conditioning programmes for work, in addition to their therapeutic exercises, which is a massive area for the occupational health service to provide. This specific type of practice provides us with the endurance needed to do our jobs.”
C6: “…sometimes physios working in occupational health departments can be seen as only doing musculoskeletal assessments, yet their workload is not just musculoskeletal, they must know our job and develop a rehabilitation programme that can help us stay in our job.”
C3: “Physiotherapists have to initiate, together with the multidisciplinary team, a suitable work programme. So it’s easy to see that their role is more than just strengthening muscles and loosening joints.”
C8: “I think it’s better getting a physio who can do work rehabilitation, which is probably better that getting a physio who can only do musculoskeletal work.”
Support for injuries at work
C1: “I think an important contribution of physiotherapists is the help they give to your care when you get injured at work. Sometimes, I feel like the managers just panic when staff is injured, and usually, they don’t know what to do, so I think it’s nice when you have an experienced physio on-site that deals with these types of injuries.”
C3: “Physiotherapists can see staff following an accident at work because I feel they will have a better idea of the injury.”
C3: “For someone who has just been injured then direct physio can help.”
C9: “They can help staff recover from sore muscles and tendons, also I think they can help with strains and disabilities so that staff member are reassured that when they are injured, somebody is there to support them.”
Improving staff health
C3: “I’m glad there is a staff physio in our hospital because I was able to recover from my health issues much quicker, and the workplace adjustments recommended helped me do my job better.”
C4: “When I told the physio about my condition, I was given an immediate appointment, which I felt was so refreshing because I did not have to go through so many different channels. The physio also contacted my GP so that my investigations could be speeded up.”
C5: “I just called the physio and I was given advice over the phone to reduce the swelling in my leg, and I was then fast-tracked to urgent care centre, and all this has definitely helped speed up my recovery.”
C4: “Frankly, the sooner someone helps you, the sooner you can return to work, and if that means getting a physiotherapist on board, then I’m all for it.”
Job coaching
C2: “The physiotherapist provides us with on-the-job coaching, which helps us to learn new techniques to do the job or adapt the job to fit in with our problems.”
Development of job descriptions
C1: “The physiotherapist is best placed to assess functions of the individual, and I think it is important they use this information to help managers develop job descriptions that are functionally based. This will help to understand what type of staff to hire so that we can get people who can do the job.”

The first theme focused on vocational rehabilitation. Clients felt that the contribution of physiotherapists in occupational health services went beyond initial assessment and treatment and incorporated functional capacity evaluations. Physiotherapists were also seen as being capable of developing and choosing appropriate tools that contributed to functional testing. Clients also claimed that physiotherapists were able to support the process of job demand analysis and to contribute to work-related modifications for injured clients. The contribution of physiotherapists to work-specific rehabilitation was perceived as embracing the rehabilitation needs of the workforce. Most clients believed that physiotherapists had relevance in providing a specialized exercise program that focused on functional aspects of their rehabilitation as opposed to general nonspecific exercises.

The second theme focused on health promotion. Clients viewed the contribution of physiotherapists as professionals that could enhance their health and well-being and to assist them recover faster so that they could perform their job tasks efficiently. Physiotherapists were also seen as a connection between clients and their managers for improving their health in the workplace. Other contributions included the involvement in developing job descriptions and supporting workplace health promotion events.

## Discussion

The study explored the perceptions of clients about the contribution of physiotherapists in occupational health services and is an important precursor to future research. Clients welcomed the contribution of occupational health physiotherapists in 2 major areas, that is, vocational rehabilitation and health promotion.

It was a small study, so it is not possible to generalize the findings to all physiotherapists working in occupational health services in the United Kingdom. Physiotherapists would need to judge for themselves the transferability of the findings to their own settings and context. The semi-structured interviews were flexibly designed so that all clients were asked similar questions while allowing for more in-depth probing on emerging topics.

In terms of vocational rehabilitation, clients’ revealed distinct components of the contribution of physiotherapists in occupational health services such as functional capacity evaluations, job demand analysis, and work specific rehabilitation. Functional capacity evaluations, while employed in some occupational health services, are currently not standard practice for all physiotherapists. The purpose of functional capacity evaluations is to provide standardized, objective and unbiased information for an employer or potential employer regarding the ability of an employee to undertake the demands of the job (6). There is evidence to suggest that individual employees underestimate their actual physical capability (7), and functional capacity evaluations, therefore, can help provide an unbiased assessment of an employee’s physical capabilities and enhance the physiotherapist’s recommendations for fitness for work, inform vocational rehabilitation programs, and supplement the advice given by occupational health physicians and nurses.

Clients also felt that physiotherapists had a role in undertaking job demand analysis. The main purpose for carrying out a job demand analysis is to accurately match the functional tests selected for the functional capacity evaluation with work-related activities to improve the validity of functional testing (8). Another benefit of using a job demand analysis prior to undertaking a functional capacity evaluation is that it provides a minimal performance criterion to undertake the job rather than assuming a better performance in the functional testing is a better predictor of work participation (8). It was further proposed that following a job demand analysis, if an employee’s performance exceeds the minimum required to carry out the job, then the employee’s capability is more likely to be sufficient to undertake it (9).

Interestingly, clients reported a role for physiotherapists in developing job descriptions. While most employers have job descriptions, these are usually very generic and do not contain the specific functional information needed (eg, standing, bending/stooping, lifting, carrying, kneeling, gripping) to meet the job requirements. For a physiotherapist working in an occupational health service to contribute to the development of job descriptions, there would need to be an evaluation of the performance of a healthy employee undertaking the same or very similar job in order to make recommendations during the job description development process.

Promoting staff health was another pertinent issue reported by clients and ensures that physiotherapists offer a holistic approach toward improving staff health. Physiotherapists working in occupational health services are in a unique position to offer holistic care because they have the flexibility to spend more time with clients to be able to manage a variety of their health and vocational needs.

Another contribution of physiotherapists was a role in job coaching. Job coaching may take the form of “on-the-job” training and has a general reputation as being the most effective method for developing vocational work because it involves the client learning at their place of work while they are engaged in the actual job. Ciampa (2005) recommended that a person with substantial coaching experience undertakes the job coaching role, which may be supported by formal classroom teaching, web-based technology, or video conferencing (10). Physiotherapists will need to clearly outline which features of the client’s job they are competent to coach, potentially even necessitating upgrading their skills or receiving formal coaching qualifications to ensure that they understand and uphold the levels of professionalism, standards, and ethics required to be a coach.

## Conclusion

Clients clearly felt that physiotherapists made a contribution to occupational health services in vocational rehabilitation and health promotion. This information lays the groundwork for the development of physiotherapists within occupational health services, which in turn will help clients achieve better care and health outcomes. Further research may need to focus on exploring the contributions of physiotherapists from a variety of other stakeholders, such as commissioners and workforce managers, and how this may subsequently impact the developing role within occupational health services.
